# Real-world risk assessment of combined cilostazol-rosuvastatin: a retrospective cohort study using Korean electronic health records

**DOI:** 10.3389/fphar.2026.1752835

**Published:** 2026-04-17

**Authors:** Woo-young Shin, Ha Young Jang, Kiyoung Lee

**Affiliations:** 1 Department of Family Medicine, Chung-ang University Gwangmyeong Hospital, Chung-Ang University College of Medicine, Seoul, Republic of Korea; 2 College of Pharmacy, Gachon University, Incheon, Republic of Korea; 3 Department of Internal Medicine, Gachon University Gil Medical Center, Gachon University School of Medicine, Incheon, Republic of Korea

**Keywords:** cilostazol, drug safety, electronic health record, pharmacovigilance, real-world evidence, rosuvastatin

## Abstract

**Background:**

Cilostazol and rosuvastatin are frequently co-prescribed for cardiovascular disease management, particularly in Asian populations. Despite widespread clinical use, comprehensive real-world safety data for this drug combination remain limited.

**Objective:**

To evaluate the 1-year safety profile of cilostazol-rosuvastatin combination therapy compared with that of cilostazol monotherapy in a real-world Korean population using electronic health records.

**Methods:**

This retrospective cohort study utilised the Observational Medical Outcomes Partnership (OMOP) Common Data Model from Gachon University Gil Hospital (2004–2025). Patients on stable rosuvastatin therapy (≥90 days) who had cilostazol added to their treatment (n = 262) were compared to cilostazol monotherapy patients (n = 6,323). Primary outcomes were incidence of atherosclerotic cardiovascular disease (ASCVD), bleeding, and myopathy at 90 and 365 days. Secondary outcomes included changes in liver enzyme levels and platelet count. Fisher’s exact and Welch’s t-tests were used for statistical analyses.

**Results:**

The combination group had higher baseline comorbidities compared with the monotherapy group, including chronic kidney disease (45.8% vs. 32.1%), diabetes mellitus (55.0% vs. 29.2%), and concurrent antithrombotic therapy (61.1% vs. 27.9%). At the 365-day follow-up, no primary safety events occurred in the combination group (0/113) versus ASCVD 2/2,307 (0.1%), bleeding 3/2,307 (0.1%), and myopathy 1/2,307 (0.0%) in the monotherapy group (p = 1.00). Laboratory parameters remained stable: AST/ALT change +14.0 vs. +5.7 U/L (p = 0.71), platelet change −8.1 vs. +18.0 ×10^9^/L (p = 0.14).

**Conclusion:**

Cilostazol-rosuvastatin combination therapy demonstrated a favourable real-world safety profile despite a higher baseline risk. These findings support the safety of this combination in routine clinical practice.

## 1 Introduction

Polypharmacy in cardiovascular disease management presents both opportunities and challenges for patient safety. Cilostazol, a selective phosphodiesterase-3 inhibitor, and rosuvastatin, a potent HMG-CoA reductase inhibitor, are frequently co-prescribed in clinical practice, particularly in East Asian countries, where cilostazol use exceeds Western prescription patterns for peripheral arterial disease and stroke prevention (Shinohara et al., 2010; Kim et al., 2018).

The pharmacological rationale for this combination therapy is compelling. Both drugs target complementary mechanisms in atherothrombosis whilst undergoing hepatic metabolism through distinct pathways. Rosuvastatin is minimally metabolised (∼10%) primarily via CYP2C9, whereas cilostazol undergoes CYP3A4-mediated metabolism, theoretically minimising drug–drug interactions Drugs.com (2024a), Drugs.com (2024b). Recent pharmacokinetic studies in healthy volunteers have demonstrated minor interactions within bioequivalence boundaries (Kim et al., 2024). Preclinical evidence suggests potential synergistic anti-inflammatory effects (Cho et al., 2019).

However, both medications carry inherent safety concerns. Statins can cause dose-related transaminase elevation in approximately 1% of patients and myopathy in 5%–10% of patients undergoing treatment (Bays, 2006; Stroes et al., 2015). A recent pharmacovigilance analysis of cilostazol identified multiple safety signals, including cardiovascular events (Huang et al., 2025); however, subsequent real-world studies have shown no increase in major adverse cardiovascular events over extended follow-up periods (Lee et al., 2020). Additionally, Asian populations demonstrate approximately two-fold higher systemic exposure to rosuvastatin than Caucasians, necessitating careful safety monitoring (Lee et al., 2005).

Despite widespread clinical use, comprehensive real-world safety data for cilostazol-rosuvastatin combination therapy remain limited. Whilst the individual drug safety profiles are well characterised, the specific safety of their combination remains understudied, particularly in populations with higher statin sensitivity. Most existing evidence derives from small pharmacokinetic studies in healthy volunteers not designed to detect clinical adverse events. Large-scale observational studies using electronic health records provide unique opportunities to evaluate drug safety under actual clinical conditions (Sherman et al., 2016).

Therefore, this study aimed to characterise the real-world safety profile of cilostazol-rosuvastatin combination therapy—prescribed as separate agents (a free combination)—using electronic health records from a Korean tertiary hospital. Given the established safety profiles of each medication, we specifically focused on whether their concurrent use exacerbates the risk of cardiovascular events, bleeding, myopathy, and laboratory parameter changes over a 1-year follow-up period.

## 2 Methods

### 2.1 Ethics approval

This study was approved by the Institutional Review Board of the Gachon University Gil Hospital (IRB No. GCIRB2025-233). The requirement for informed consent was waived owing to the retrospective nature of the de-identified data. The study followed the principles of the Declaration of Helsinki and the STROBE guidelines for observational studies.

### 2.2 Study design and data source

This retrospective cohort study used electronic health records from Gachon University Gil Hospital, a 1,400-bed tertiary teaching hospital in Incheon, South Korea. Data were extracted from the hospital’s clinical data warehouse and converted to the Observational Medical Outcomes Partnership (OMOP) Common Data Model (CDM) version 5.3. The study period spanned from January 2004 to July 2025. OMOP CDM standardisation ensures data quality and facilitates reproducible research through consistent terminology and structures.

### 2.3 Study population

Two cohorts were identified: (1) combination therapy group–patients on stable rosuvastatin therapy (≥90 days) who had cilostazol newly added, and (2) monotherapy group–patients newly prescribed cilostazol without rosuvastatin exposure. We deliberately implemented a new-user design for both cohorts. Excluding prevalent (chronic) combination users minimizes inherent pharmacoepidemiologic biases, such as survivor bias (depletion of susceptibles) and immortal time bias, ensuring that the early hazard periods following treatment initiation are accurately captured (Ray, 2003; Suissa, 2008). The index date was defined as the first day of concurrent drug administration in the combination group and the first cilostazol prescription date in the monotherapy group. Concurrent use required ≥4 days of overlapping prescription periods, accounting for drug half-lives (cilostazol ∼11–13 h, rosuvastatin ∼19 h) (Drugs.com, 2024a; Drugs.com, 2024b).

Exclusion criteria were as follows: baseline diagnosis of atherosclerotic cardiovascular disease (ASCVD), bleeding disorders, myopathy before the index date, and any prescription history of statins other than rosuvastatin.

### 2.4 Outcomes and statistical analysis

Primary safety outcomes were incident ASCVD (composite of myocardial infarction, stroke, and cardiovascular death), bleeding events, and myopathy, assessed at 90- and 365-day post-index on an on-treatment basis. These specific endpoints were chosen because they represent the most severe, clinically significant adverse events historically associated with cilostazol and rosuvastatin, thereby allowing us to capture any potential synergistic harm. Secondary outcomes included changes in liver enzyme levels (AST/ALT) and platelet count.

We acknowledge potential confounding factors by indication, as clinicians preferentially prescribed combination therapy to high-risk patients. Incidence rates per 100 person-years were estimated using Poisson exact methods, including exact confidence intervals even when event counts were zero. Adjusted hazard ratios (HRs) were obtained from multivariable Cox proportional hazards models including age at index date, sex, history of diabetes mellitus, chronic kidney disease, hypertension, baseline use of antiplatelet and anticoagulant therapy. Kaplan-Meier survival curves were generated to visually assess time-to-event outcomes. Welch’s *t*-test was used for laboratory changes. Baseline characteristics were compared using standardised mean differences (SMD >0.1, indicating a significant imbalance).

Post-hoc power calculation: there were 262 and 6,323 patients in the combination and monotherapy groups, respectively. The study had 80% power to detect relative risk ≥2.5 for adverse events (α = 0.05, β = 0.20). Given multiple comparisons, primary outcomes were considered confirmatory, whilst secondary outcomes were exploratory. Analyses were performed using R version 4.3.0.

## 3 Results

### 3.1 Baseline characteristics

From the 19,798 cilostazol and 51,976 rosuvastatin prescriptions, 27,776 patients with prior non-rosuvastatin statin use and 258 patients with baseline exclusion diagnoses were excluded. The final cohort comprised 262 combination therapy patients and 6,323 monotherapy patients.

The combination group had substantially higher baseline comorbidities, including chronic kidney disease (45.8% vs. 32.1%, SMD = 0.28), diabetes mellitus (55.0% vs. 29.2%, SMD = 0.54), and concurrent antithrombotic therapy (61.1% vs. 27.9%, SMD = 0.70) (Table 1). A detailed profile of concomitant medications, including specific antiplatelets and anticoagulants, is provided in Supplementary Table S1. Median age was 62.2 vs. 64.5 years (SMD = 0.12). The median cilostazol dose was 100 mg/d (IQR 100–200) in both groups, and the rosuvastatin dose in the combination group was 10 mg/d (IQR 10–10). Treatment persistence was longer in the combination therapy group than in the monotherapy group (median, 317 vs. 97 days).

**TABLE 1:** Baseline characteristics of the study cohort.

Variable	Combination group (n = 262)	Monotherapy group (n = 6,323)	SMD*
Demographics			
Age, mean (SD), years	62.2 (12.1)	64.5 (13.8)	0.12
Female, n (%)	129 (49.3)	3,035 (48.0)	0.03
Baseline laboratory values, mean (SD)			
AST/ALT (U/L)	28.00 (14.38)	24.09 (12.03)	0.30
Total bilirubin (mg/dL)	0.65 (0.25)	0.67 (0.26)	0.08
CK (U/L)	25.41 (36.80)	63.86 (108.66)	0.48
FPG (mg/dL)	109.50 (19.09)	121.18 (36.06)	0.40
eGFR (mL/min/1.73m^2^)	64.75 (22.37)	76.87 (27.28)	0.49
Platelet count (×10^9^/L)	207.94 (54.01)	232.39 (66.97)	0.40
Comorbidities, n (%)			
Chronic kidney disease	120 (45.8)	2,031 (32.1)	0.28
Diabetes mellitus	144 (55.0)	1,846 (29.2)	0.54
Hypertension	23 (8.8)	292 (4.6)	0.17
Medication characteristics			
Cilostazol dose, median (IQR), mg/day	100 (100–200)	100 (100–200)	-
Rosuvastatin dose, median (IQR), mg/day	10 (10–10)	N/A	-
Cilostazol treatment duration, median (IQR), days	317 (94–847)	97 (30– 293)	-
Concomitant medications, n (%)			
Any antithrombotic agents	160 (61.1)	1,767 (27.9)	0.70
Antiplatelet agents	151 (57.6)	1,628 (25.7)	0.68
Anticoagulant agents	60 (22.9)	325 (5.1)	0.51
Fibrates	8 (3.1)	82 (1.3)	0.12
Other cardiovascular drugs†	185 (70.6)	2,892 (45.7)	0.52

* SMD, standardised mean difference; values >0.1 indicate a meaningful imbalance.

^†^ Includes ACE, inhibitors; ARBs, beta-blockers, calcium channel blockers.

N/A, not applicable; NR, not reported; IQR, interquartile range.

Complete follow-up data were available for 171/262 (65.3%) and 3,543/6,323 (56.0%) patients in the combination and monotherapy groups at 90 days, and 113/262 (43.1%) and 2,307/6,323 (36.5%) at 365days, respectively.

### 3.2 Primary safety outcomes

During the 365-day follow-up period, no primary safety events occurred in the combination group (0/113, 0.0% for ASCVD, bleeding, and myopathy, respectively) (Table 2). The monotherapy group demonstrated ASCVD 2/2,307 (0.1%), bleeding 3/2,307 (0.1%), myopathy 1/2,307 (0.0%). Multivariable Cox analyses demonstrated no evidence of increased clinical risk associated with initiating cilostazol following chronic rosuvastatin therapy (Table 3; Supplementary Figure S1). Bleeding events did not occur in the combination group, resulting in an adjusted hazard ratio (HR) approximating zero (HR 0.00, 95% CI 0.00–Inf). Myopathy likewise showed no cases in the combination cohort (HR 0.00, 95% CI 0.00–Inf). For the ASCVD composite outcome, the risk was not significantly different between groups, with an adjusted HR of 0.94 (95% CI 0.43–2.04). Overall, across all three prespecified outcomes—bleeding, myopathy, and ASCVD—the combination group did not exhibit any safety or cardiovascular disadvantage compared with cilostazol monotherapy.

**TABLE 2:** Primary and secondary safety outcomes.

Outcome	Follow-up period	Combination group	Monotherapy group	p-value
Primary safety outcomes, n/N (%)				
ASCVD				
​	≥90 days	0/171 (0.0)	4/3,543 (0.1)	1.00
​	≥365 days	0/113 (0.0)	2/2,307 (0.1)	1.00
Bleeding				
​	≥90 days	0/171 (0.0)	5/3,543 (0.1)	1.00
​	≥365 days	0/113 (0.0)	3/2,307 (0.1)	1.00
Myopathy				
​	≥90 days	0/171 (0.0)	1/3,543 (0.0)	1.00
​	≥365 days	0/113 (0.0)	1/2,307 (0.0)	1.00
Composite primary outcome				
​	≥90 days	0/171 (0.0)	10/3,543 (0.3)	1.00
​	≥365 days	0/113 (0.0)	6/2,307 (0.3)	1.00
Secondary outcomes: Laboratory changes				
Mean change from baseline (SD)				
AST/ALT (U/L)				
​	≥90 days	−7.3 (10.6)	+0.41 (26.3)	0.30
​	≥365 days	+14.0 (31.0)	+5.7 (39.3)	0.71
Total bilirubin (mg/dL)				
​	≥90 days	−0.05 (0.1)	−0.01 (0.2)	0.91
​	≥365 days	N/A	−0.1 (0.3)	-
Platelet count (×10^9^/L)				
​	≥90 days	−10.6 (59.6)	+11.5 (48.8)	0.32
​	≥365 days	−8.1 (73.7)	+18.0 (55.4)	0.14
Clinical significance thresholds				
AST/ALT >2× ULN or >3× baseline				
​	≥90 days	0/171 (0.0)	Data not available	-
​	≥365 days	0/113 (0.0)	Data not available	-
Platelet count <100 ×10^9^/L				
​	≥90 days	0/171 (0.0)	Data not available	-
​	≥365 days	0/113 (0.0)	Data not available	-
Follow-up data availability, n/N (%)				
Complete primary outcome data				
​	≥90 days	171/262 (65.3)	3,543/6,323 (56.0)	-
​	≥365 days	113/262 (43.1)	2,307/6,323 (36.5)	-
Complete laboratory data				
​	≥90 days	Variable by parameter*	Variable by parameter*	-
​	≥365 days	Limited availability†	Limited availability†	-

ASCVD, atherosclerotic cardiovascular disease; ULN, upper limit of normal; N/A, not available; NR, not reported.

* Laboratory data availability varies by parameter, with liver function tests having the highest completion rates.

^†^The 365-day laboratory data showed substantial attrition, limiting the comparative analysis of some parameters.

**TABLE 3:** Comparative risk of bleeding, myopathy, and cardiovascular events: adjusted hazard ratios.

Outcome	Incidence rate per 100 PY*	Incidence rate per 100 PY*	Adjusted HR \† (95% CI)	p-value
Bleeding	0.00 (0.00–0.81)	0.25 (0.17–0.37)	0.00 (0.00–Inf)	0.997
Myopathy	0.00 (0.00–0.81)	0.07 (0.03–0.14)	0.00 (0.00–Inf)	0.998
ASCVD	2.36 (1.08–4.48)	0.80 (0.63–0.99)	0.94 (0.43–2.04)	0.88

* Incidence rates and 95% CIs, are from Poisson exact methods (per 100 person-years).

^†^ Adjusted hazard ratios from Cox proportional hazards models with monotherapy group as the reference group. Models were adjusted for age at index date, sex, history of diabetes mellitus, chronic kidney disease, hypertension, baseline use of antiplatelet and anticoagulant therapy.

### 3.3 Secondary outcomes: laboratory parameters

Changes in liver enzyme levels remained clinically insignificant. At 365 days, mean AST/ALT change was +14.0 U/L (combination) vs. +5.7 U/L (monotherapy), p = 0.71. Platelet count changes: −8.1 vs. +18.0 ×10^9^/L, p = 0.14. None of the patients met criteria for clinically significant abnormalities (AST/ALT >3× baseline or platelet count <100 × 10 units/L). Laboratory data availability varied by parameter; creatine kinase, glucose, and eGFR measurements were limited, precluding reliable comparative analyses.

## 4 Discussion

This real-world cohort study provides evidence regarding the safety of cilostazol-rosuvastatin combination therapy in clinical practice. Despite substantial baseline differences between the groups, with the combination cohort having a higher prevalence of chronic kidney disease (45.8% vs. 32.1%), diabetes mellitus (55.0% vs. 29.2%), and concurrent antithrombotic use (61.1% vs. 27.9%), no major adverse events occurred during the 1-year follow-up. The absence of ASCVD events, bleeding complications, and myopathy in the combination group, along with stable laboratory parameters, support the clinical feasibility of this therapeutic approach.

These findings align with the pharmacological basis for safety, as distinct metabolic pathways (CYP2C9 for rosuvastatin, and primarily CYP3A4 and CYP2C19 for cilostazol) minimise interaction potential (Drugs.com, 2024a; Drugs.com, 2024b). These results are particularly relevant for East Asian populations, where cilostazol is commonly prescribed (Shinohara et al., 2010; Kim et al., 2018) and rosuvastatin exposure is predicted to increase (Lee et al., 2005).

Important limitations of this study include the observational design, with inherent confounding by indication (Psaty et al., 1999), as evidenced by the preferential combination therapy prescribed to higher-risk patients. Although we rigorously adjusted for measured confounders using multivariable Cox regression, residual confounding may remain. The study’s limited statistical power (80% to detect RR ≥ 2.5) and single-centre setting may affect the generalisability of the findings. Additionally, the relatively small combination cohort (n = 262) and missing laboratory data for certain parameters hinder definitive conclusions regarding rare adverse events.

Furthermore, several specific methodological constraints must be acknowledged. First, retrospective EHR data cannot systematically capture subjective intolerances (e.g., mild headache) or routine bleeding times; however, the markedly shorter treatment duration in the monotherapy group likely reflects such early intolerances leading to discontinuation in real-world practice. Second, to address the substantial 365-day attrition and unequal follow-up, we utilised an on-treatment analysis and time-to-event right-censoring models, which inherently mitigate potential discontinuation bias. Third, our $\ge 4$-day overlap definition was based on steady-state pharmacokinetics, but the limited sample size precluded sensitivity analyses with longer overlap windows. Finally, the median rosuvastatin dose in our combination cohort was 10 mg/d. Therefore, these findings should be cautiously extrapolated to patients receiving high-intensity rosuvastatin or those with advanced renal impairment.

Nevertheless, this study addresses a significant gap in available evidence by providing real-world safety data for commonly prescribed drug combinations. These findings can inform clinical decision making regarding cardiovascular polypharmacy, although individual patient assessments remain essential. Larger multicentre studies are warranted to confirm these findings and explore the optimal patient selection criteria for combination therapy.

## Data Availability

The raw data supporting the conclusions of this article will be made available by the authors, without undue reservation.
