# Supplementary material for: Real-world risk assessment of combined cilostazol-rosuvastatin: a retrospective cohort study using Korean electronic health records
Source: Front Pharmacol. 2026 Apr 17;17:1752835. doi: 10.3389/fphar.2026.1752835 (PMC13133040; doi:10.3389/fphar.2026.1752835)

Supplementary Table 1

Supplementary Table 1. Concomitant Medication Profiles of the Study Cohort

Drug Class	Drug	Combination (n=262)	Monotherapy (n=6,323)
Antiplatelets	Aspirin	150 (57.3%)	1,618 (25.6%)
	Clopidogrel	110 (41.9%)	531 (8.4%)
	Prasugrel	3 (1%)	0 (0%)
	Ticagrelor	1 (0.3%)	0 (0%)
	Abciximab	0 (0%)	6 (0.1%)
	Tirofiban	0 (0%)	0 (0%)
Anticoagulants	Heparin	55 (21.0%)	297 (4.7%)
	Enoxaparin	1 (0.3%)	13 (0.2%)
	Dalteparin	0 (0%)	13 (0.2%)
	Argatroban	0 (0%)	0 (0%)
	Apixaban	3 (1%)	25 (0.4%)
	Dabigatran	0 (0%)	6 (0.1%)
	Edoxaban	0 (0%)	0 (0%)
	Rivaroxaban	0 (0%)	13 (0.2%)
	Warfarin	3 (1%)	57 (0.9%)
Lipid-lowering	Fenofibrate	8 (3.2%)	82 (1.3%)

Supplementary Figure 1

Supplementary Figure 1. Kaplan–Meier Curves for Bleeding, Myopathy, and ASCVD Outcomes in the Study Cohort

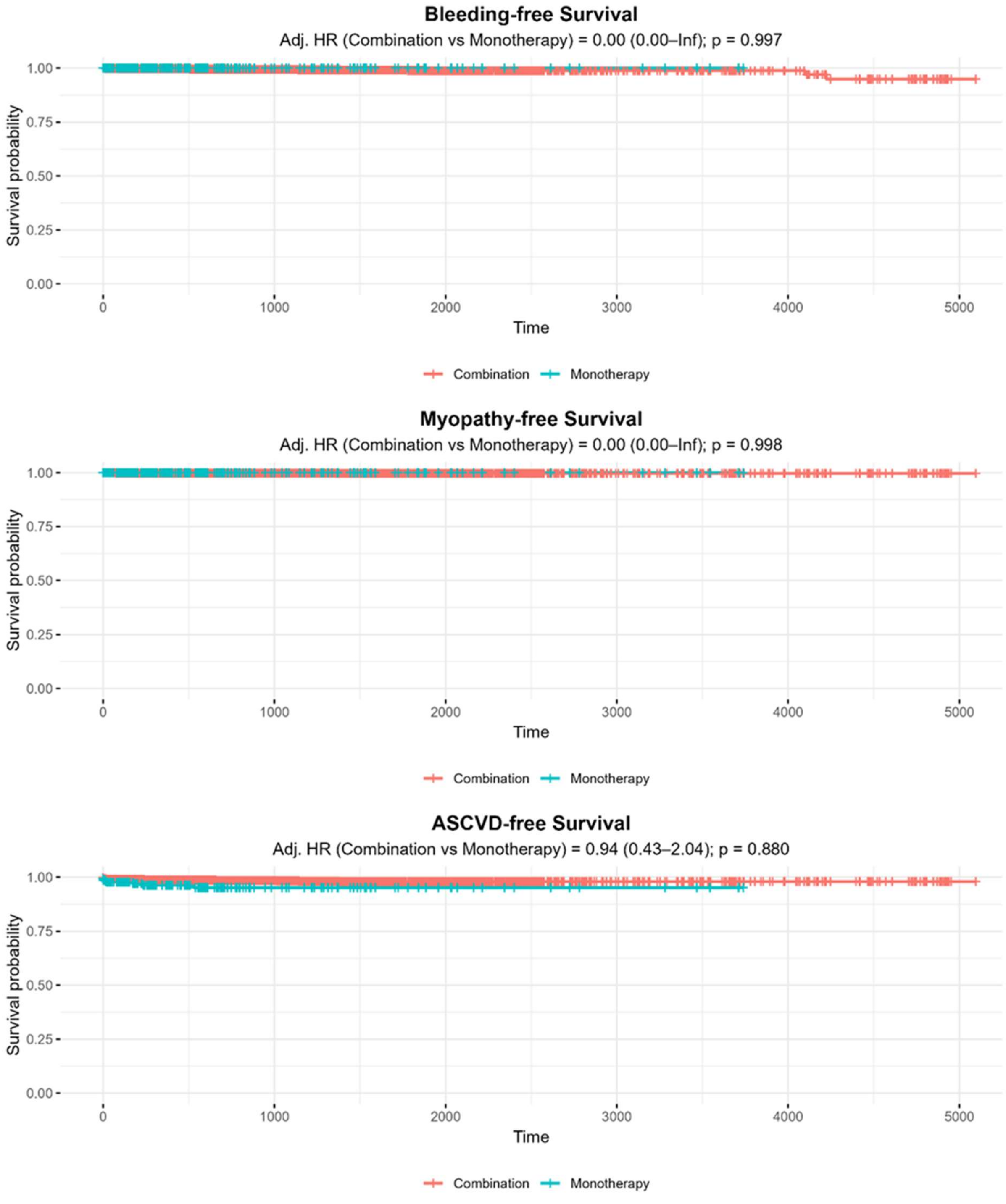

Supplement: Supplementary file 1 [file DataSheet1.pdf]
